# Supplementary material for: Transcranial magnetic stimulation (TMS) inhibits cortical dendrites
Source: eLife. 2016 Mar 18;5:e13598. doi: 10.7554/eLife.13598 (PMC4811769; doi:10.7554/eLife.13598)
Supplement: Figure 1—source data 1. — DOI: http://dx.doi.org/10.7554/eLife.13598.004 [file elife-13598-fig1-data1.pdf]

Source data: Figure 1

	Integral (ΔF/F * ms)		Amplitude (ΔF/F)			
			1st component		2nd component	
	HS	HS+TMS	HS	HS+TMS	HS	HS+TMS
1	6417.40798	4491.94593	4.48152	3.35724	5.37158	3.87253
2	4250.9649	2916.89863	3.98382	3.59984	5.18377	4.72779
3	2140.39701	1595.39302	3.48996	2.67636	3.21066	3.18638
4	18269.43596	10910.19211	22.7865	17.756	17.0107	15.5202
5	4782.63198	2076.24575	4.76702	3.684	5.73755	5.96591
6	4994.04079	4304.98143	7.7	6.6	7.4	5.8
7	4183.666547	2991.411615	4.63491	3.72264	5.58804	3.91326
8	5401.886305	1471.961019	4.86384	1.86808	6.10376	2.82619
9	3065.697341	1727.26411	3.45045	1.81816	4.67765	3.06919
10	1402.012191	888.488839	3.20611	2.13741	2.41401	1.70993
11	3030.7	1321.54	3	1.7	3.89	2
12	3169.07	538.37	4.60802	2.63728	4.92847	3.43839
13	972.23	764.8	1.43	0.22232	2.366	1.85246
14	2867.96	1059.43	3.92	2.98	5.85	5.21
15	3317.30055	1004.26866	17.6275	11.3076	7.56435	4.25859
16	3787.45685	2134.49819	14.5968	7.85005	4.8308	3.19071
17	840.282479	378.18018	15.0552	10.6564	10.5944	8.30207
